# Variation in lung function and alterations in cardiac structure and function—Analysis of the UK Biobank cardiovascular magnetic resonance imaging substudy

**DOI:** 10.1371/journal.pone.0194434

**Published:** 2018-03-20

**Authors:** Ross J. Thomson, Nay Aung, Mihir M. Sanghvi, Jose Miguel Paiva, Aaron M. Lee, Filip Zemrak, Kenneth Fung, Paul E. Pfeffer, Alexander J. Mackay, Tricia M. McKeever, Elena Lukaschuk, Valentina Carapella, Young Jin Kim, Charlotte E. Bolton, Stefan K. Piechnik, Stefan Neubauer, Steffen E. Petersen

**Affiliations:** 1 William Harvey Research Institute, NIHR Biomedical Research Centre at Barts, Queen Mary University of London, London, United Kingdom; 2 Barts Health NHS Trust, London, United Kingdom; 3 Nottingham Respiratory Research Unit, NIHR Nottingham Biomedical Research Centre, School of Medicine, City Hospital NUH Trust Campus, University of Nottingham, Nottingham, United Kingdom; 4 Division of Cardiovascular Medicine, Radcliffe Department of Medicine, University of Oxford, Oxford, United Kingdom; Kurume University School of Medicine, JAPAN

## Abstract

**Background:**

Reduced lung function is common and associated with increased cardiovascular morbidity and mortality, even in asymptomatic individuals without diagnosed respiratory disease. Previous studies have identified relationships between lung function and cardiovascular structure in individuals with pulmonary disease, but the relationships in those free from diagnosed cardiorespiratory disease have not been fully explored.

**Methods:**

UK Biobank is a prospective cohort study of community participants in the United Kingdom. Individuals self-reported demographics and co-morbidities, and a subset underwent cardiovascular magnetic resonance (CMR) imaging and spirometry. CMR images were analysed to derive ventricular volumes and mass. The relationships between CMR-derived measures and spirometry and age were modelled with multivariable linear regression, taking account of the effects of possible confounders.

**Results:**

Data were available for 4,975 individuals, and after exclusion of those with pre-existing cardiorespiratory disease and unacceptable spirometry, 1,406 were included in the analyses. In fully-adjusted multivariable linear models lower FEV_1_ and FVC were associated with smaller left ventricular end-diastolic (−5.21ml per standard deviation (SD) change in FEV_1_, −5.69ml per SD change in FVC), end-systolic (−2.34ml, −2.56ml) and stroke volumes (−2.85ml, −3.11ml); right ventricular end-diastolic (−5.62ml, −5.84ml), end-systolic (−2.47ml, −2.46ml) and stroke volumes (−3.13ml, −3.36ml); and with lower left ventricular mass (−2.29g, −2.46g). Changes of comparable magnitude and direction were observed per decade increase in age.

**Conclusions:**

This study shows that reduced FEV_1_ and FVC are associated with smaller ventricular volumes and reduced ventricular mass. The changes seen per standard deviation change in FEV_1_ and FVC are comparable to one decade of ageing.

## Introduction

Respiratory disease is common and under-diagnosed, and a significant and growing cause of death and disability[1]. Much of the excess morbidity and mortality is secondary to cardiovascular disease[2]. In a prospective cohort study of 7,575 patients with chronic obstructive pulmonary disease (COPD) from Saskatchewan, Canada[3], the quintile of patients with the most severe COPD had relative risks between 1.4 and 3.1 for all-cause mortality, cardiovascular mortality, congestive heart failure, and angina.

Lung function is independently associated with cardiovascular morbidity and mortality[4,5], both in individuals with established respiratory disease and in those without any diagnosis or symptoms. In a prospective study of 15,000 individuals in the west of Scotland[6], those in the lowest quintile for forced expiratory volume in first second (FEV_1_) had an all-cause mortality almost twice that of the highest quintile. The risks were similar in asymptomatic (subclinical) individuals.

The prevalence of subclinical impaired lung function is great: in the National Health and Nutrition Examination Survey (NHANES) and NHANES III cohort studies of community volunteers in the United States[7] around 12–13% of individuals had an FEV_1_ to forced vital capacity (FVC) ratio less than 0.7, the generally accepted cut-off for obstructive lung disease[8]. Almost three quarters of these individuals did not have a diagnosis of lung disease, and most of the undiagnosed individuals reported good or excellent health. Mortality was higher in those with abnormal spirometry, irrespective of the presence of diagnosed lung disease.

Previous work has explored some of the relationships between lung disease and cardiovascular structure. Using data from the MESA cohort, Grau and colleagues[9] demonstrated that increasing emphysema severity, quantified with thoracic computed tomography, is associated with lower right ventricular volumes and mass. They also found that right ventricular mass is associated with the FEV_1_ to FVC ratio. Barr *et al*.[10] continued this analysis to identify similar relationships between emphysema severity and left ventricular volumes and mass, and between the FEV_1_ to FVC ratio and left ventricular end-diastolic and stroke volumes. These analyses were conducted in an all-comers population, many of whom had existing cardiovascular and respiratory disease. Furthermore, the effects of FEV_1_ and FVC *per se* were not studied. Given the prevalence of subclinical changes in lung function, together with the associations with adverse outcomes, we set out to explore the relationships between lung function and cardiovascular structure and function in a population free from diagnosed cardiovascular and respiratory disease.

## Methods

UK Biobank is a large prospective cohort study of approximately 500,000 unselected community volunteers aged 40 to 69 at the time of enrolment, living in the United Kingdom. The design and conduct of the study have been described in detail previously[11]. This study was covered by the general ethical approval for UK Biobank studies from the NHS National Research Ethics Service on 17^th^ June 2011 (Ref 11/NW/0382). None of the authors had direct contact with the study participants. This report is a cross-sectional analysis of the subset of participants who took part in the imaging pilot programme.

Demographics and doctor-diagnosed co-morbidities were self-reported by electronic questionnaire and interview with a healthcare professional. Data collected during the imaging visit were used in the analyses, except where unavailable, in which case data from the enrolment visit were used. If a participant did not answer a question regarding a comorbid diagnosis, or did not know, it was assumed they did not have the condition. Physical measurements (height, weight, blood pressure, heart rate) and smoking status were captured exclusively at the time of imaging.

Smokers were defined as individuals who smoke, or used to smoke, on all or most days. Those who smoked occasionally were deemed to be never smokers. For current or previous smokers, pack year history was calculated as the product of the number of packs of cigarettes smoked per day and the difference between age started smoking and age stopped smoking (or age at imaging for current smokers).

### Cardiovascular magnetic resonance

A subgroup of participants is undergoing cardiovascular magnetic resonance imaging (CMR). The CMR acquisition protocol and post-processing have been described previously[12]. In brief, participants underwent imaging using a 1.5 Tesla Siemens MAGNETOM Aera scanner (Siemens Healthcare GmbH, Erlangen, Germany) at a central imaging centre. Short and long axis cine images were acquired using a balanced steady state free precession sequence. Manual image analysis was performed across two core imaging centres using cvi42 version 5.1.1 (Circle Cardiovascular Imaging, Calgary, Canada) by observers blinded to all clinical information. The software used these contours to calculate right and left ventricular end-diastolic, end-systolic, and stroke volumes; right and left ventricular ejection fraction; and left ventricular mass. The manual image analysis and quality control, including assessment of intra- and inter-observer variability, have been described in detail previously[13].

### Spirometry

Spirometry without bronchodilator administration was performed at the time of imaging according to a standard protocol using a Vitalograph Pneumotrac 6800 spirometer [14]. Each participant produced two blows, and a third if there was unacceptable variance in the first two (as calculated by the spirometer). The forced expiratory volume in one second (FEV_1_), forced vital capacity (FVC), and an automated assessment of measurement quality were recorded.

Spirometry blows were excluded from the analysis if the automated quality assessment was anything other than ‘acceptable’. Participants were excluded if they had fewer than two acceptable blows, if the coefficient of variation for the two or three acceptable blows exceeded 5%, or if the difference between the best and second best acceptable blow exceeded 150ml, in accordance with established guidelines[8]. To investigate the potential impact of these exclusion criteria on the results we performed a sensitivity analysis in which all participants with at least two ‘acceptable’ spirometry blows were included, without any limit on the permissible range or coefficient of variation between blows. The results of this sensitivity analysis are presented in S2 File. Obstructive spirometry was defined as an FEV_1_ to FVC ratio less than 0.7.

### Statistical analysis

The CMR-derived parameters and FEV_1_ and FVC were approximately normally distributed and the assumptions for linear regression were satisfied. FEV_1_ and FVC were standardised to the mean (FEV_1_ mean 2.87 litres, SD 0.70 litres; FVC mean 3.73 litres, SD 0.89 litres).

The relationships between nine CMR-derived parameters and both FVC and FEV_1_ were modelled with multivariable linear regression, using age, sex, ethnicity, height, weight, systolic blood pressure, resting heart rate, Townsend deprivation index (a commonly used measure of material deprivation where positive values represent above-average deprivation and negative values below-average deprivation), education level (categorised as the presence or absence of a degree or professional qualification), regular alcohol consumption (defined as three or more occasions per week), smoking history (pack years) and any diagnosis of hypertension or diabetes as co-variates. Height and weight were included as covariates in the regression models, rather than indexing the dependent variables to body surface area, since the use of ratios in regression analysis is liable to spurious results and misinterpretation[15,16]. The approach adopted ensures all variables in the model are appropriately adjusted for body composition.

To place the effects of spirometry on CMR-derived parameters in context, the relationships between the CMR-derived parameters and age were modelled in a similar fashion, except that both FEV_1_ and FVC were used as co-variates. Interaction terms were used to explore any stratification by sex in the relationships between the CMR-derived parameters and the primary exposure variable (FEV_1_, FVC, or age) in each regression model.

Each regression analysis was performed on a complete-case basis without imputation of missing data. Nine of thirteen covariates had no missing data, and the covariate with most missing data (alcohol consumption) lacked fewer than one percent of observations. The outcome variables (CMR-derived parameters) had fewer than two percent missing observations, which were clustered in 22 participants who were missing all the outcome variables and another one who was missing right ventricular parameters.

Regression coefficients are presented as the change in the CMR-derived parameter per standard deviation change in FEV_1_ or FVC, or per decade change in age. P values were calculated using Student’s t test or ANOVA for continuous variables and Chi-squared test for categorical variables. Statistical analyses were performed using *R* version 3.3.2[17].

## Results

**Fig 1:**
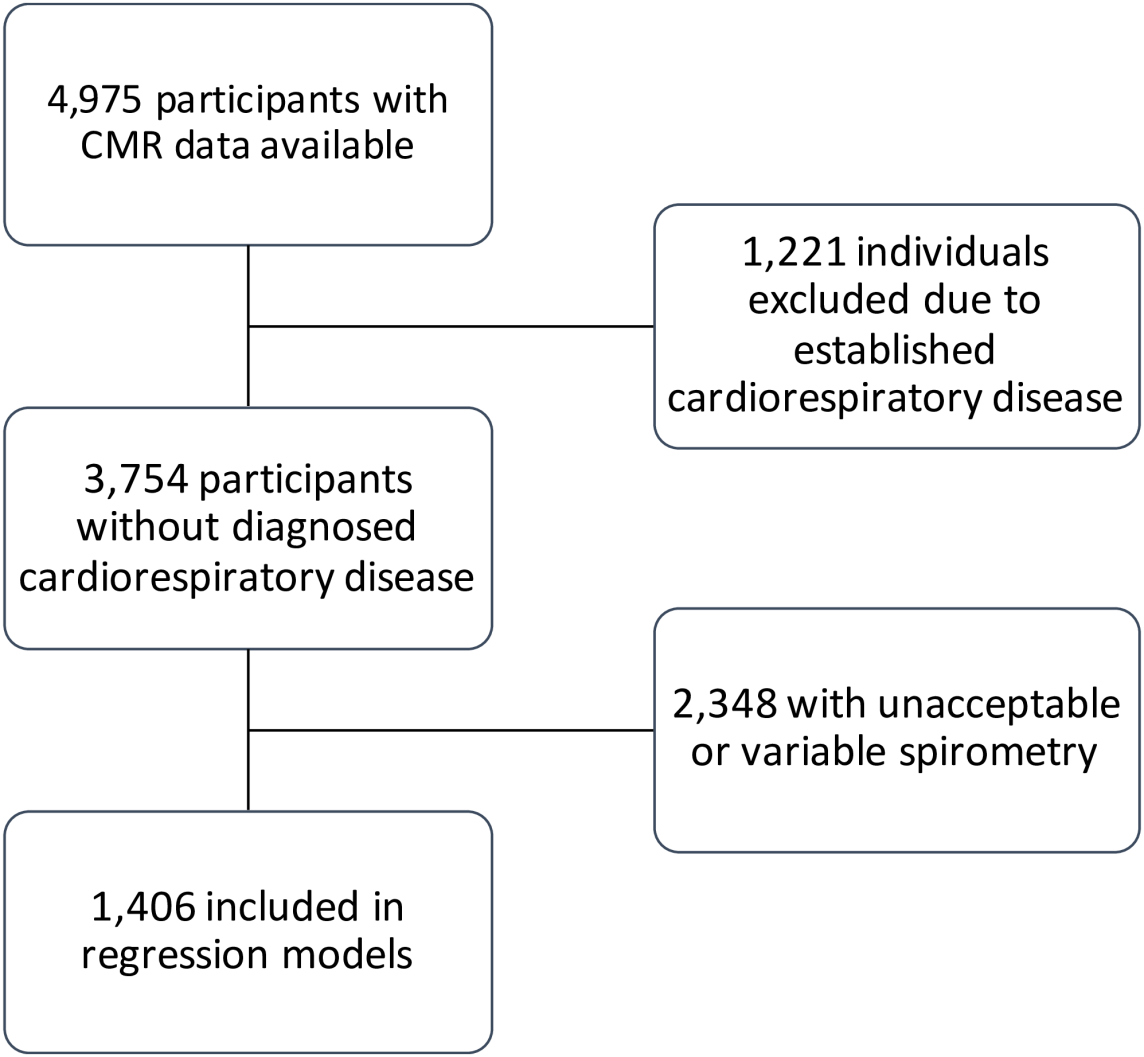
Case selection flowchart for the primary analysis.

1,221 individuals were excluded on account of pre-existing cardiorespiratory disease, the definition of which is provided in S1 File. Of the remaining 3,754 individuals, a further 2,348 were excluded in the primary analysis because they did not meet the criteria for reproducible and acceptable spirometry. The case selection process for the primary analysis is shown in Fig 1.

1,406 participants were included in the primary analysis. On account of missing data, the number of observations in the regression models was 1,366 or 1,367 depending on the CMR-derived parameter being studied. Baseline characteristics, stratified by tertile of FEV_1_, are described in Table 1. Compared to those in the highest tertile, those in the lowest tertile of FEV_1_ were older, more likely to be female, shorter, lighter, had lower diastolic blood pressure and higher resting heart rates, and were more likely to have obstructive spirometry.

**Table 1:** Baseline characteristics of the study population by tertile of FEV_1_.

	Tertile of FEV_1_			P value
	1^st^ (n = 475)	2^nd^ (n = 468)	3^rd^ (n = 463)	
Age (years)	57.6 (6.6)	53.8 (7.8)	52.3 (7.5)	< 0.001
Sex (male)	41 (9%)	162 (35%)	396 (86%)	< 0.001
Height (cm)	163 (7)	168 (7)	177 (7)	< 0.001
Weight (kg)	68 (13)	73 (15)	82 (13)	< 0.001
Diastolic BP (mmHg)	78 (10)	78 (10)	80 (9)	0.002
Systolic BP (mmHg)	137 (19)	135 (18)	137 (16)	0.976
Resting heart rate (beats per minute)	72 (10)	69 (11)	68 (12)	< 0.001
Townsend deprivation index	-1.99 (2.76)	-2.01 (2.66)	-1.84 (2.75)	0.388
Hypertension	128 (27%)	130 (28%)	121 (26%)	0.852
Diabetes	22 (5%)	15 (3%)	22 (5%)	0.423
Obstructive spirometry^a^	63 (13%)	34 (7%)	19 (4%)	< 0.001
Smoking history (pack years)	4.71 (10)	4.87 (11)	5.23 (11)	0.448
Educational level (degree or professional qualification)	312 (66%)	304 (65%)	314 (68%)	0.632
Ethnicity (white)	456 (97%)	457 (98%)	455 (98%)	0.428
Alcohol consumption (three or more drinks per week)	88 (19%)	87 (19%)	98 (21%)	0.524

Data represent mean (standard deviation) or n (percentage) for continuous and categorical variables, respectively.

The cut-offs between the first and second and second and third tertiles, of FEV_1_ were 2.5 litres and 3.09 litres, respectively.

P values by ANOVA or Chi-squared test.

^a^Obstructive spirometry defined as an FEV_1_ to FVC ratio < 0.7.

Following adjustment for potential confounders in a multivariable linear model, lower FEV_1_ and FVC were associated with smaller left ventricular (LV) end-diastolic volume, LV end-systolic volume, LV stroke volume, right ventricular (RV) end-diastolic volume, RV end-systolic volume, RV stroke volume, and LV mass (Table 2). The interaction terms between the primary exposure variables and sex were not statistically significant and thus there were no differences in the observed relationships between males and females. The linear relationships between CMR-derived parameters and FEV_1_ and FVC are shown in the S1 and S2 Figs, respectively.

**Table 2:** Effects of lung function on CMR-derived parameters.

CMR Parameter	FEV_1_ (standardised)				FVC (standardised)			
	Effect estimate	95% CI		P value	Effect estimate	95% CI		P value
		Lower	Upper			Lower	Upper	
Left ventricular end-diastolic volume (ml)	−5.21	−7.42	−3.00	< 0.001	−5.69	−8.03	−3.36	< 0.001
Left ventricular end-systolic volume (ml)	−2.34	−3.78	−0.89	0.002	−2.56	−4.09	−1.03	0.001
Left ventricular stroke volume (ml)	−2.85	−4.22	−1.49	< 0.001	−3.11	−4.55	−1.67	< 0.001
Left ventricular mass (g)	−2.29	−3.77	−0.82	0.002	−2.46	−4.02	−0.89	0.002
Left ventricular ejection fraction (%)	NS	−0.55	0.60	0.927	NS	−0.56	0.65	0.886
Right ventricular end-diastolic volume (ml)	−5.62	−7.98	−3.26	< 0.001	−5.84	−8.34	−3.34	< 0.001
Right ventricular end-systolic volume (ml)	−2.47	−4.03	−0.92	0.002	−2.46	−4.10	−0.82	0.003
Right ventricular stroke volume (ml)	−3.13	−4.50	−1.76	< 0.001	−3.36	−4.81	−1.91	< 0.001
Right ventricular ejection fraction (%)	NS	−0.68	0.48	0.739	NS	−0.78	0.44	0.588

Effect sizes represent the change of the CMR parameter per standard deviation reduction in FEV_1_ or FVC in a multivariable linear regression adjusted for age, sex, ethnicity, height, weight, systolic blood pressure, resting heart rate, Townsend deprivation index, education level, regular alcohol consumption, smoking history, and any diagnosis of hypertension or diabetes. CI; confidence interval. NS; not statistically significant.

**Fig 2:**
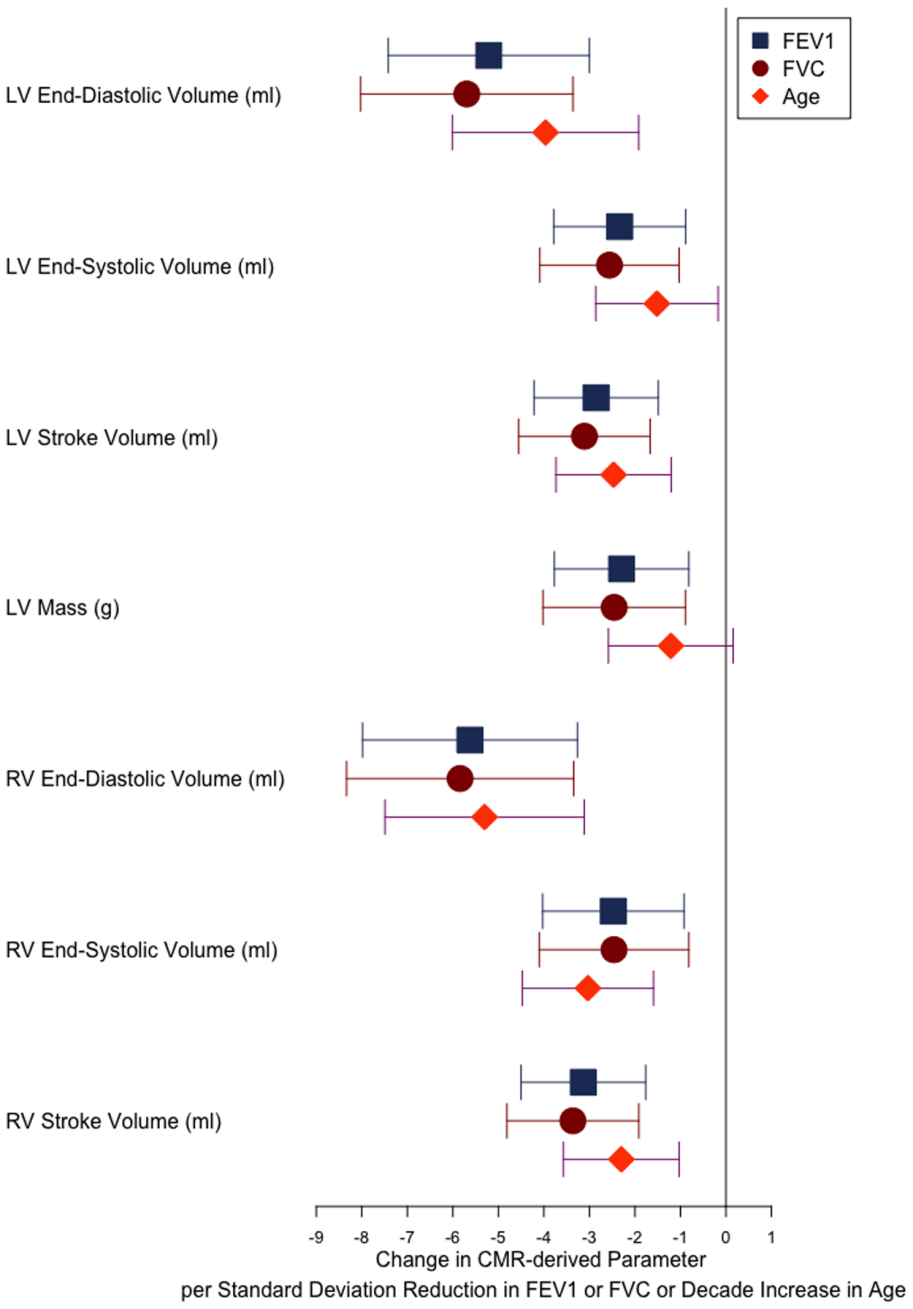
Effect sizes for the change in CMR-derived parameter per standard deviation reduction in FEV_1_ and FVC, and per one decade increase in age. Filled shapes represent the change in the CMR-derived parameter per standard deviation reduction in FEV_1_ or FVC, or per decade increase in age. Error bars represent the 95% confidence interval for the effect estimate.

In a similar multivariable linear model, increasing age was associated with smaller LV end-diastolic volume (−3.96ml, −6.01ml to −1.92ml), LV end-systolic volume (−1.52ml, −2.86ml to −0.17ml, LV stroke volume (−2.47ml, −3.73ml to −1.20ml), RV end-diastolic volume (−5.30ml, −7.49ml to −3.11ml), RV end-systolic volume (−3.03ml, −4.47ml to −1.59ml), and RV stroke volume (−2.30ml, −3.57ml to −1.03ml) (Table 3). Values represent the mean and lower and upper 95% confidence intervals for the change in CMR-derived parameter per decade increase in age. FEV_1_, FVC, and age did not influence right or left ventricular ejection fraction. Age did not influence left ventricular mass. The relative effect sizes of FEV_1_, FVC, and age on CMR-derived parameters are shown in Fig 2.

**Table 3:** Effects of age on CMR-derived parameters.

CMR Parameter	Age (decades)			
	Effect estimate	95% CI		P value
		Lower	Upper	
Left ventricular end-diastolic volume (ml)	−3.96	−6.01	−1.92	< 0.001
Left ventricular end-systolic volume (ml)	−1.52	−2.86	−0.17	0.027
Left ventricular stroke volume (ml)	−2.47	−3.73	−1.20	< 0.001
Left ventricular mass (g)	NS	−2.58	0.16	0.083
Left ventricular ejection fraction (%)	NS	−0.39	0.67	0.614
Right ventricular end-diastolic volume (ml)	−5.30	−7.49	−3.11	< 0.001
Right ventricular end-systolic volume (ml)	−3.03	−4.47	−1.59	< 0.001
Right ventricular stroke volume (ml)	−2.30	−3.57	−1.03	< 0.001
Right ventricular ejection fraction (%)	NS	−0.13	0.94	0.142

Effect sizes represent the change of the CMR parameter per decade increase in age in a multivariable linear regression adjusted for FEV_1_, FVC, sex, ethnicity, height, weight, systolic blood pressure, resting heart rate, Townsend deprivation index, education level, regular alcohol consumption, smoking history, and any diagnosis of hypertension or diabetes. CI; confidence interval. NS; not statistically significant

In a sensitivity analysis employing a broader definition of acceptable spirometry (at least two ‘acceptable’ blows with no restriction on the range or coefficient of variation of these blows) 2,070 individuals met the criteria for inclusion. There were minimal changes to the effect sizes in the multivariable linear regression, and the 95% confidence intervals were narrower on account of the larger sample size (S2 File). There were no changes to the direction or statistical significance of the results.

## Discussion

The key finding of our study is that in a large cohort of individuals without prior diagnosis of cardiorespiratory disease, lower lung function is associated with smaller left and right ventricular end-systolic, end-diastolic, and stroke volumes; and with lower left ventricular mass. These relationships are independent of other variables known to affect cardiac structure. There was no association with ejection fraction.

The results of our study confirm and expand on previous work. The Multi Ethnic Study of Atherosclerosis (MESA) lung substudy has previously identified relationships between emphysema severity, quantified by thoracic computed tomography, and right and left ventricular volumes and mass[9,10,18], although the consistency of the relationships varied between analyses, and many of the patients had diagnoses of cardiovascular and respiratory disease. In the same cohort, reduced FEV_1_ to FVC ratio was associated with LV end-diastolic volume and LV stroke volume[10], and with RV mass[9] although associations with FEV_1_ and FVC *per se* were not studied. Using echocardiography, Watz and colleagues[19] showed that LV end-diastolic diameter and RV diameter are associated with FEV_1_. Our study extends these previous findings to a cohort without diagnosed heart or lung disease, and demonstrates consistent relationships between spirometry and multiple measures of left and right ventricular structure and function. The effect sizes seen in this study (2.29ml to 5.84ml per standard deviation change in FEV_1_ or FVC) are comparable to those seen in MESA[10] and in echocardiographic studies[19]. Furthermore, they are comparable in size to the effects of systolic and diastolic blood pressure, and diabetes, all widely accepted drivers of ventricular remodelling[20].

Age is a significant risk factor for cardiovascular morbidity and mortality[21,22], and is associated with the development of ventricular fibrosis, remodelling, and diastolic dysfunction[23]. Previous investigations have explored the relationship between age and ventricular structure and function. Analysis of a subset of the UK Biobank CMR study, free from all reported comorbid disease[13] showed a statistically significant negative correlation between age and LV and RV end-diastolic, end-systolic, and stroke volumes; and LV mass. Similar relationships were found using the MESA dataset[24,25], and in the current study population.

In the current study, the variation in ventricular volumes and mass seen with lower FEV_1_ and FVC is comparable to that seen with ageing. Notably, the changes in CMR-derived parameters per standard deviation of FEV_1_ and FVC are approximately the same as those seen with one decade of ageing. This suggests lower lung function is associated with a ‘premature ageing’ effect on the ventricle. This provides potential insight into the mechanisms responsible for adverse cardiovascular outcomes in those with deranged lung function, and highlights the potential importance of measuring lung function as a marker of cardiovascular ‘ageing’ and risk.

Our analysis did not identify any relationship between ejection fraction and FEV_1_, FVC, or age, despite the significant influence of these factors on other measures of ventricular function. Similar results were found in a MESA analysis of individuals with emphysema[10]. Our results add to the evidence that ejection fraction alone is an insensitive marker of cardiac function and remodelling, particularly as it relates to respiratory function.

This study has several limitations. The design of UK Biobank renders it liable to selection bias, and it is likely that the population recruited to the study is not completely representative of the population as a whole. The self-reporting of co-morbidities and lifestyle factors such as smoking is liable to ascertainment bias. The relatively low prevalence of smoking observed in our study population may be explained, at least in part, by these two factors.

Many participants were excluded from the analysis as their spirometry did not meet conventional criteria for reproducibility and validity. It is possible that the variability in spirometry arose from its acquisition in the non-specialist UK Biobank assessment centre, rather than in a dedicated pulmonary function laboratory. Furthermore, the study protocol limited participants to producing three blows, rather than repeating the measurement until valid and reproducible results were achieved, as is commonly done in clinical practice. Nonetheless, even after individuals with unacceptable variation had been excluded the full range of FEV_1_ and FVC were represented, and thus the ability to examine relationships between these parameters and cardiac structure was preserved. Furthermore, the sensitivity analysis revealed that exclusion of these individuals did not materially affect the results, and suggests that no significant selection bias was introduced by the exclusion of individuals with unacceptable spirometry. The inclusion only of individuals with reproducible, high quality spirometry increases the confidence in the relationships identified by this study.

This study raises a number of questions worthy of further analysis, including the long term cardiovascular outcomes of individuals with subclinical changes in lung function, the changes in cardiovascular phenotype over time in those with deranged spirometry, and evaluation of ventricular fibrosis in participants with impaired lung function through T1 mapping. These will be amenable to investigation as longitudinal follow-up and parametric mapping data become available from UK Biobank.

## Conclusions

In a large cohort of patients without known cardiorespiratory disease, lower FEV_1_ and FVC are associated with smaller left and right ventricular volumes, and lower left ventricular mass. The changes in ventricular structure per standard deviation fall in FVC and FEV_1_ are similar to those seen with a one decade increase in age, and may shed light on the mechanisms underlying increased cardiovascular risk in those with subclinical changes in lung function, as well as the importance of lung function as a risk factor for cardiovascular disease.

## Supporting information

S1 File Definitions of existing cardiorespiratory disease. (DOCX)

S2 File Sensitivity analysis exploring the impact of a less restrictive definition of valid and reproducible spirometry that included all participants with at least two ‘acceptable’ blows (as determined by the spirometer) without limitation on the coefficient of variation or difference between the best and second best blow. (DOCX)

S1 Fig Univariable associations between CMR-derived parameters and FEV_1_. Each panel shows the association between one CMR-derived parameter and FEV_1_ prior to the standardisation of the lung function. R^2^ is the coefficient of explained variance, calculated as the square of the Pearson correlation coefficient between the CMR-derived parameter and FEV_1_ on a complete pairs basis.
(TIF)

S2 Fig Univariable associations between CMR-derived parameters and FVC. Each panel shows the association between one CMR-derived parameter and FVC prior to standardisation of the lung function. R^2^ is the coefficient of explained variance, calculated as the square of the Pearson correlation coefficient between the CMR-derived parameter and FVC on a complete pairs basis.
(TIF)
