# Supplementary material for: Variation in lung function and alterations in cardiac structure and function—Analysis of the UK Biobank cardiovascular magnetic resonance imaging substudy
Source: PLoS One. 2018 Mar 20;13(3):e0194434. doi: 10.1371/journal.pone.0194434 (PMC5860758; doi:10.1371/journal.pone.0194434)
Supplement: S1 File — (DOCX) [file pone.0194434.s001.docx]

**S1 File**

**Definitions of existing cardiorespiratory disease**

Existing cardiovascular or respiratory disease was defined as any self-reported or doctor’s diagnosis of pulmonary embolism or deep vein thrombosis, emphysema or chronic bronchitis, asthma, interstitial lung disease, pulmonary fibrosis, sleep apnoea, respiratory failure, chronic obstructive pulmonary disease, bronchiectasis, fibrosing alveolitis, asbestosis, myocardial infarction, cardiomyopathy, heart failure, arrhythmia, valve disease, atrial fibrillation, pericardial disease, stroke or transient ischaemic attack, and peripheral vascular disease. This list encompasses all of the terms related to cardiovascular and respiratory disease used by participants when asked for their comorbidities.
