# Supplementary material for: Variation in lung function and alterations in cardiac structure and function—Analysis of the UK Biobank cardiovascular magnetic resonance imaging substudy
Source: PLoS One. 2018 Mar 20;13(3):e0194434. doi: 10.1371/journal.pone.0194434 (PMC5860758; doi:10.1371/journal.pone.0194434)
Supplement: S2 File — (DOCX) [file pone.0194434.s002.docx]

**S2 File**

**Sensitivity analysis**

The application of commonly used criteria for determining the validity and reproducibility of spirometry (coefficient of variation < 5% and difference between the best and second best blow < 150ml) resulted in 2,348 participants being excluded from the primary analysis. To assess for any bias introduced by these criteria we performed a sensitivity analysis in which any participant with at least two ‘acceptable’ blows (as defined by the spirometer’s automated quality control) were included, with no restriction on the coefficient of variation or range. 2,070 participants were included in this sensitivity analysis.

Table A. Effects of lung function on CMR-derived parameters with broader criteria for spirometry

|  | **FEV_1_** (standardised) | | | | **FVC** (standardised) | | | |
| --- | --- | --- | --- | --- | --- | --- | --- | --- |
|  | Effect estimate | 95% CI | | P value | Effect estimate | 95% CI | | P value |
| **CMR Parameter** |  | Lower | Upper |  |  | Lower | Upper |  |
| Left ventricular end-diastolic volume (ml) | −4.11 | −6.01 | −2.22 | < 0.001 | −4.95 | −6.95 | −2.95 | < 0.001 |
| Left ventricular end-systolic volume (ml) | −1.62 | −2.88 | −0.36 | 0.012 | −1.90 | −3.23 | −0.57 | 0.005 |
| Left ventricular stroke volume (ml) | −2.49 | −3.62 | −1.37 | < 0.001 | −3.05 | −4.25 | −1.85 | < 0.001 |
| Left ventricular mass (g) | −2.46 | −3.65 | −1.28 | < 0.001 | −2.51 | −3.77 | −1.25 | < 0.001 |
| Left ventricular ejection fraction (%) | NS | −0.34 | 0.59 | 0.602 | NS | −0.39 | 0.59 | 0.695 |
| Right ventricular end-diastolic volume (ml) | −5.49 | −7.42 | −3.55 | < 0.001 | −6.13 | −8.17 | −4.09 | < 0.001 |
| Right ventricular end-systolic volume (ml) | −2.81 | −4.09 | −1.53 | < 0.001 | −2.91 | −4.26 | −1.55 | < 0.001 |
| Right ventricular stroke volume (ml) | −2.66 | −3.78 | −1.54 | < 0.001 | −3.21 | −4.39 | −2.02 | < 0.001 |
| Right ventricular ejection fraction (%) | NS | −0.33 | 0.61 | 0.571 | NS | −0.47 | 0.52 | 0.917 |

Effect sizes represent the change of the CMR parameter per standard deviation reduction in FEV_1_ or FVC in a multivariable linear regression adjusted for age, sex, ethnicity, height, weight, systolic blood pressure, resting heart rate, Townsend deprivation index, education level, regular alcohol consumption, smoking history, and any diagnosis of hypertension or diabetes. CI; confidence interval. NS; not statistically significant.
