# Supplementary figures and images for: Variation in lung function and alterations in cardiac structure and function—Analysis of the UK Biobank cardiovascular magnetic resonance imaging substudy
Source: PLoS One. 2018 Mar 20;13(3):e0194434. doi: 10.1371/journal.pone.0194434 (PMC5860758; doi:10.1371/journal.pone.0194434)

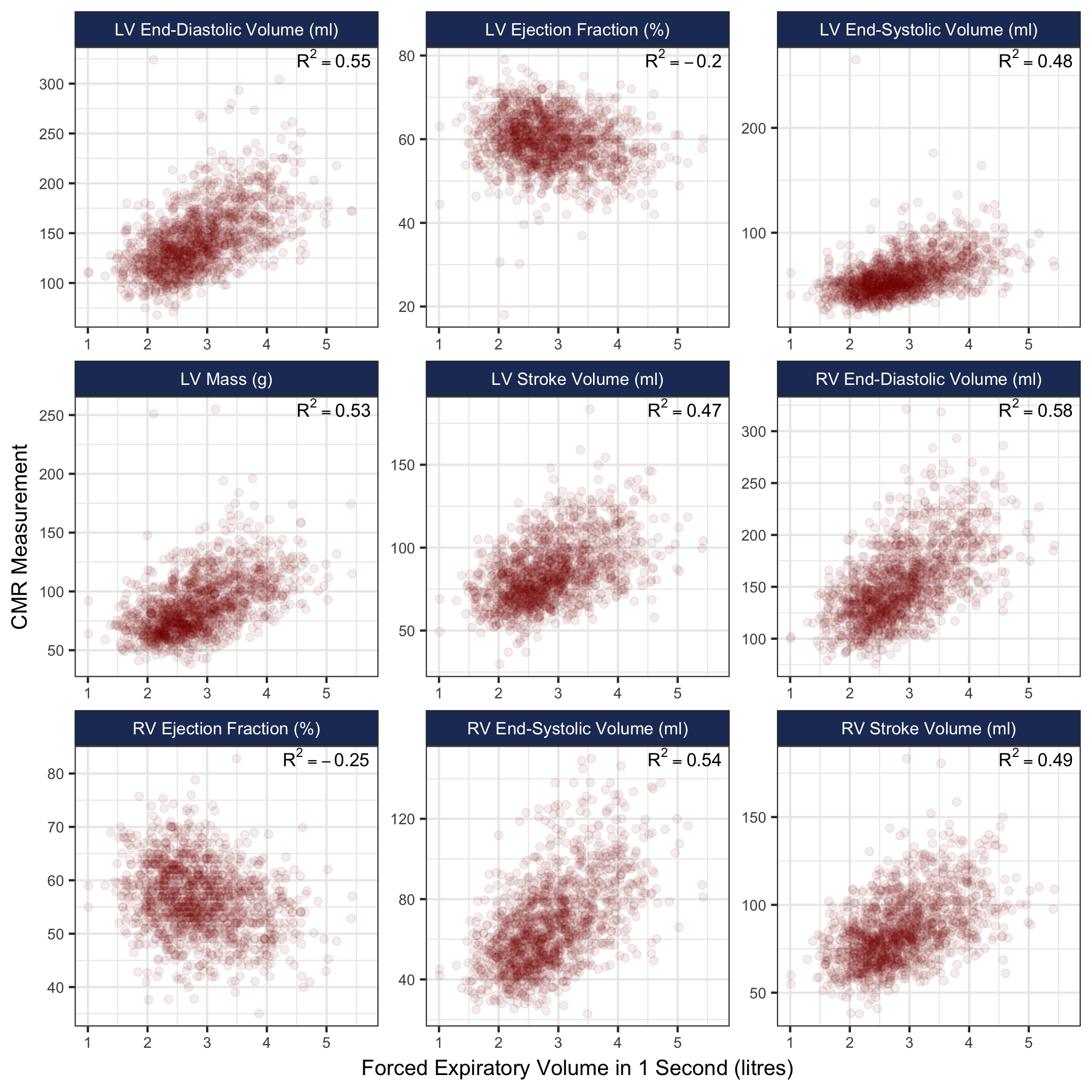

Supplement: S1 Fig — Each panel shows the association between one CMR-derived parameter and FEV1 prior to the standardisation of the lung function. R2 is the coefficient of explained variance, calculated as the square of the Pearson correlation coefficient between the CMR-derived parameter and FEV1 on a complete pairs basis. (TIF) [file pone.0194434.s003.tif]

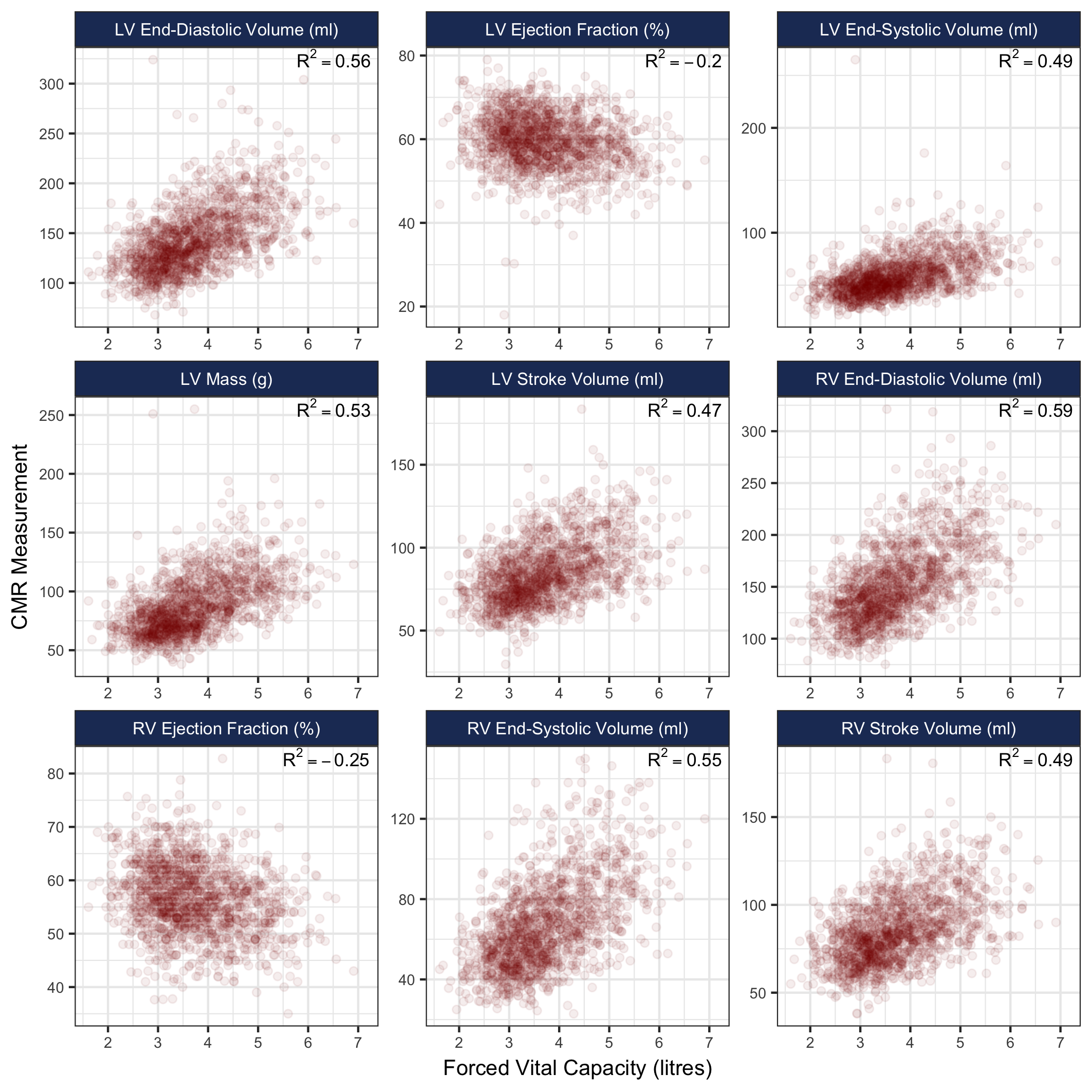

Supplement: S2 Fig — Each panel shows the association between one CMR-derived parameter and FVC prior to standardisation of the lung function. R2 is the coefficient of explained variance, calculated as the square of the Pearson correlation coefficient between the CMR-derived parameter and FVC on a complete pairs basis. (TIF) [file pone.0194434.s004.tif]
